# Post-cementation sensitivity of bridges cemented with different adhesive resin cements

**DOI:** 10.4317/jced.62513

**Published:** 2025-03-01

**Authors:** Diaaeldin Farag, Abdelrahman Almufti, Mohamad Alghalayini, Nasser Shaheen, Mahmoud Darwish, Sherif Sadek

**Affiliations:** 1 BDS, MSc, PhD, Associate Professor of Prosthodontics, Department of Restorative Dental Sciences, Gulf Medical University, Ajman, UAE. Department of Crown & Bridge, Faculty of Dentistry, Suez Canal University, Ismailia, Egypt; 2 BDS, MSD Prosthodontics. Image Dental Clinic, Riyadh, Saudi Arabia; 3 MSc Student Department of Endodontics, Faculty of Dentistry King Abdulaziz University, Saudi Arabia; 4 Professor of Prosthodontics, Restorative and Prosthetic Dental Sciences Department, Dar Al Uloom University, Riyadh, Saudi Arabia; 5 Associate Professor of Prosthodontics. Department of Prosthodontics, Vision College, Riyadh, KSA. Department of Prosthodontics, Faculty of Dentistry, Suez Canal University, Ismailia, Egypt; 6 Associate Professor of Prosthodontics. Department of Restorative Dental Sciences, Gulf Medical University, Ajman, UAE. Associate Professor Prosthodontic Department, Faculty of Dentistry, Cairo University, Egypt

## Abstract

**Background:**

Post-cementation sensitivity remains a common clinical concern in fixed partial dentures (FPDs), with variations in adhesive resin cement formulations influencing patient discomfort and long-term success. This study aimed to compare post-cementation sensitivity in FPDs cemented with three different resin cements: self-adhesive resin cements, resin cement with a self-etch adhesive, and resin cement with an etch-and-rinse adhesive.

**Material and Methods:**

A total of 27 patients demanding the replacement of one missing posterior tooth with a FPD were selected for the study. Participants were randomly assigned to one of four groups based on the type of cement used. The abutments, which were caries- and restoration-free, were prepared using standard techniques. In total, 68 abutment teeth were prepared across all patients, of which 65 were vital abutments. The FPDs were cemented using one of the following cements: Multilink Speed (MUS) (self-adhesive resin cement), Rely-X Unicem (RXU) (self-adhesive resin cement), Rely-X Ultimate & Single Bond Universal (RXUS) (self-etch adhesive mode), and Rely-X Ultimate & Single Bond Universal (RXUE) (etch-and-rinse mode). Sensitivity to cold water, air and biting was assessed pre-operatively as a control and post-operatively at 24 hours, 2 weeks, and 6 weeks using a visual analogue scale (VAS). The data were analysed using the Mann-Whitney U test.

**Results:**

Sensitivity to cold decreased significantly for most cements within 2 to 6 weeks, although VAS scores for RXUE remained above the 30% level. RXUE also exhibited significantly higher sensitivity to biting compared to Multilink Speed (MUS), Rely-X Unicem (RXU), and Rely-X Ultimate in self-etch mode (RXUS). MUS showed no biting sensitivity, while RXU and RXUS displayed mean values below 5% at the 2-week mark.

**Conclusions:**

The use of self-adhesive resin cements (MUS and RXU) and the resin cement with self-etch adhesive (RXUS) resulted in significantly lower post-operative sensitivity compared to the resin cement with etch-and-rinse adhesive (RXUE) in FPDs. Clinical Application: When cementing FPDs, particularly in cases involving freshly cut dentin, it is advisable to use self-adhesive cements or resin cements with self-etch adhesive.

** Key words:**Post-cementation sensitivity, self-adhesive, etch-and-rinse, self-etch, resin cement.

## Introduction

The introduction of self-adhesive resin cement in 2004 marked a significant advancement in prosthodontic therapy, simplifying the cementing process by eliminating the need for separate etching, priming, and bonding steps. This type of cement offers easy application and good bond strength for various restorations. Resin cement with self-etch adhesives integrate etching and bonding in a single step, reducing post-operative sensitivity and providing strong adhesion with a moderate level of etching, which is particularly suitable for sensitive teeth. Conversely, resin cements with etch-and-rinse adhesives involve a distinct etching step before application, delivering the highest bond strength, ideal for complex restorative scenarios. Each type of cement is used to specific clinical conditions, offering distinct advantages in terms of ease of use, bond strength, and moisture tolerance, which are critical for achieving durable and aesthetically pleasing results in restorative dentistry (1).

Post-operative sensitivity has been linked to the use of various types of cements, particularly resin-based ones. Clinical research has shown differing rates of post-cementation sensitivity depending on the type of restoration used. For example, a practice-based study with 210 patients, which utilized two glass ionomer-based cements to bond porcelain-fused-to-metal crowns, found that half of the participants experienced some level of sensitivity to temperature and pressure, with mean sensitivity scores of 0.52 and 0.23, respectively, on a scale of 0-10. The other half of the participants reported no sensitivity at all (2). In a clinical study conducted by Yoneda *et al*. (3), which examined vital teeth used as abutments for fixed partial dentures, as well as inlay, onlay, and crown restorations, found no instances of post-cementation sensitivity in any of the patients.

Post-operative sensitivity can result from various factors, including the choice of cement (such as zinc phosphate, glass ionomer, or resin), the degree of tooth preparation (4,5), issues with provisional restorations (6), the use of smear layer removal agents (7) and the presence of occlusal discrepancies. In a study by Balaji A *et al*. (8) the effects of polymerization in resin-modified glass ionomer cement and dual-cure resin cement were compared in relation to their crystalline structures, and these findings were correlated with clinical post-operative sensitivity. The study revealed that dual-cure resin cement generated significantly higher lattice strain than resin-modified glass ionomer cement. However, this increased strain did not correspond to higher post-operative sensitivity in dual-cure resin cements.

There is limited research available in the current literature on post-operative sensitivity, particularly following cementation. Thus, the aim of this study is to evaluate post-cementation sensitivity to cold and biting in fixed partial dentures (FPDs) cemented with two types of self-adhesive resin cements, and to compare these results with adhesive resin cements used in combination with both etch-and-rinse and self-etch systems. The null hypothesis for this study proposes that no significant difference exists in post-cementation sensitivity among the cements being tested.

## Material and Methods

The research protocol for this clinical study received approval from the research committee at Vision Colleges, ensuring ethical considerations in the use of human subjects (IRB No.: alf. dent-2020061). The study recruited patients needing posterior fixed partial dentures (FPDs) to replace a single missing tooth, who presented at the dental clinics at Vision Colleges between 2020 and 2022. The study was designed to be double-blinded, meaning neither the patients nor the evaluators were aware of which resin cement was used.

Specific inclusion criteria were followed for patient recruitment:

• Patients were either in good health or had non-significant medical conditions.

• The abutment teeth were located in the posterior region.

• Abutments were vital, free from caries, restorations, and symptoms.

• Patients taking analgesics and anti-inflammatory drugs were excluded.

• Patients with super-erupted and/or mesially tilted mandibular molar abutments were excluded.

• Individuals who had undergone periodontal surgery within the last three months were not considered.

• Pregnant women were excluded.

• Patients had to have completed at least a high school level education.

Patients meeting these criteria were verbally informed about the study’s objectives, and those willing to be involved were asked to sign a consent form outlining the aim, nature, and timeline of the study, including required recall appointments.

Pre-operative bitewing and periapical radiographs were taken to ensure the abutment teeth had adequate bone support. The abutments had a normal occlusion with the opposing teeth. Sensitivity to air iced water, and biting was assessed for each abutment before starting the procedure. The abutments were prepared for porcelain-fused-to-metal (PFM) retainers with 1.5-2 mm occlusal reduction, 1.2 labial/buccal finish line and 0.5 mm lingual finish line. A high-speed handpiece with water/air spray and diamond burs was used for the preparation.

To control bleeding and expose preparation margins, the gingival tissue was retracted using a medicated cord (Ultra Pack cord, South Jordan, USA). Impressions were made with an addition silicone elastomeric material (Hydrorise, Zhermack, Spa, USA) using a custom acrylic tray. A mono-phase technique was employed. Temporary bridges (Protemp, 3M/ESPE, Seefeld, Germany) were fabricated using a putty index matrix and cemented with non-eugenol provisional cement (Temp Bond, Kerr, USA).

All prostheses were fabricated at a single laboratory. At the cementation appointment, local anaesthesia was administered, provisional crowns were removed, and the abutments were cleaned with a pumice slurry and a rubber cup. Participants were randomly assigned to one of four resin cements: 1) Rely-Ultimate in etch-and-rinse mode (RXUE) with Scotch bond etchant and Single Bond adhesive (3M/ESPE, St. Paul, USA), 2) Rely-Ultimate in self-etch mode (RXUS) with Single Bond adhesive, 3) Rely-X Unicem (RXU) self-adhesive resin cement (3M/ESPE, Seefeld, Germany), and 4) Multilink Speed (MUS) self-adhesive resin cement (Ivoclar Vivadent, Schaan, Liechtenstein).

In addition to baseline measurements (control), hypersensitivity was evaluated by a single operator: Twenty-four hours, two weeks, and six weeks after cementation. During each visit, the abutment’s sensitivity to biting, air exposure, and cold-water exposure was assessed, following the criteria outlined by Johnson *et al*. (12).

To test sensitivity to biting, participants were asked to bite with firm pressure on the cotton tipped applicator placed on the abutment’s central fossa and rate their sensitivity on a visual analogue scale (VAS) from 0 to 10, where 0 indicated “no sensitivity” and 10 indicated “the highest level of sensitivity.” Cold water and air sensitivity were also tested. A polyvinyl siloxane stent was made to isolate the tested abutment from the rest of the teeth in the same arch. The tested abutment was exposed by creating a hole in the stent, and 5 cm³ of ice water was applied for 5 seconds using a plastic syringe. The participant then recorded their sensitivity using the VAS, and the test was stopped immediately if severe pain was experienced. A stream of air was directed at the facial surface of the crown towards the margin using an air-water syringe, while another stent with a facial opening was positioned in place. After, these tests, participants recorded their sensitivity level.

One day after cementation, participants, assisted by the clinician, completed questionnaires documenting any sensitivity they had experienced since the insertion of the FPD. Chair-side sensitivity tests were repeated at: twenty-four hours, two weeks, and six weeks post-cementation. The data was analyzed statistically using the Mann-Whitney U test, with a 95% confidence level (*P* < 0.05).

## Results

The study included 27 participants, consisting of 15 females and 12 males, with ages ranging from 30 to 40 years. A total of 65 mandibular and maxillary posterior abutment teeth were used, comprising 31 molars and 34 premolars. The distribution of the cements was as follows: MUS cement was applied to 20 retainers (11 premolars and 9 molars), RXU to 18 retainers (9 premolars and 9 molars), RXUE to 12 retainers (6 premolars and 6 molars), and RXUS to 15 retainers (8 premolars and 7 molars).

 Table 1 presents the means and standard deviations of VAS scores for different sensations (cold, biting, and air) at various intervals for the four cements. RXUE exhibited substantially higher average VAS scores in comparison to RXU, MUS, and RXUS across all tests at each time point (*p* < 0.05). Additionally, RXUE had exhibited substantially higher average VAS scores in comparison to the pretreatment control for all three tests, even after six weeks (*p* < 0.05).

No statistically significant differences were observed in the mean VAS scores between RXU, MUS and RXUS across the three tests at any time intervals (*p* > 0.05). For the cold test, Both RXU and MUS showed higher mean VAS scores that is statistically significant compared to pretreatment values: (*p* < 0.05) for RXU and (*p* < 0.001) for MUS after 24 hours. However, this significant difference was no longer present after two weeks for RXU and after six weeks for MUS.

**Figure 1:**
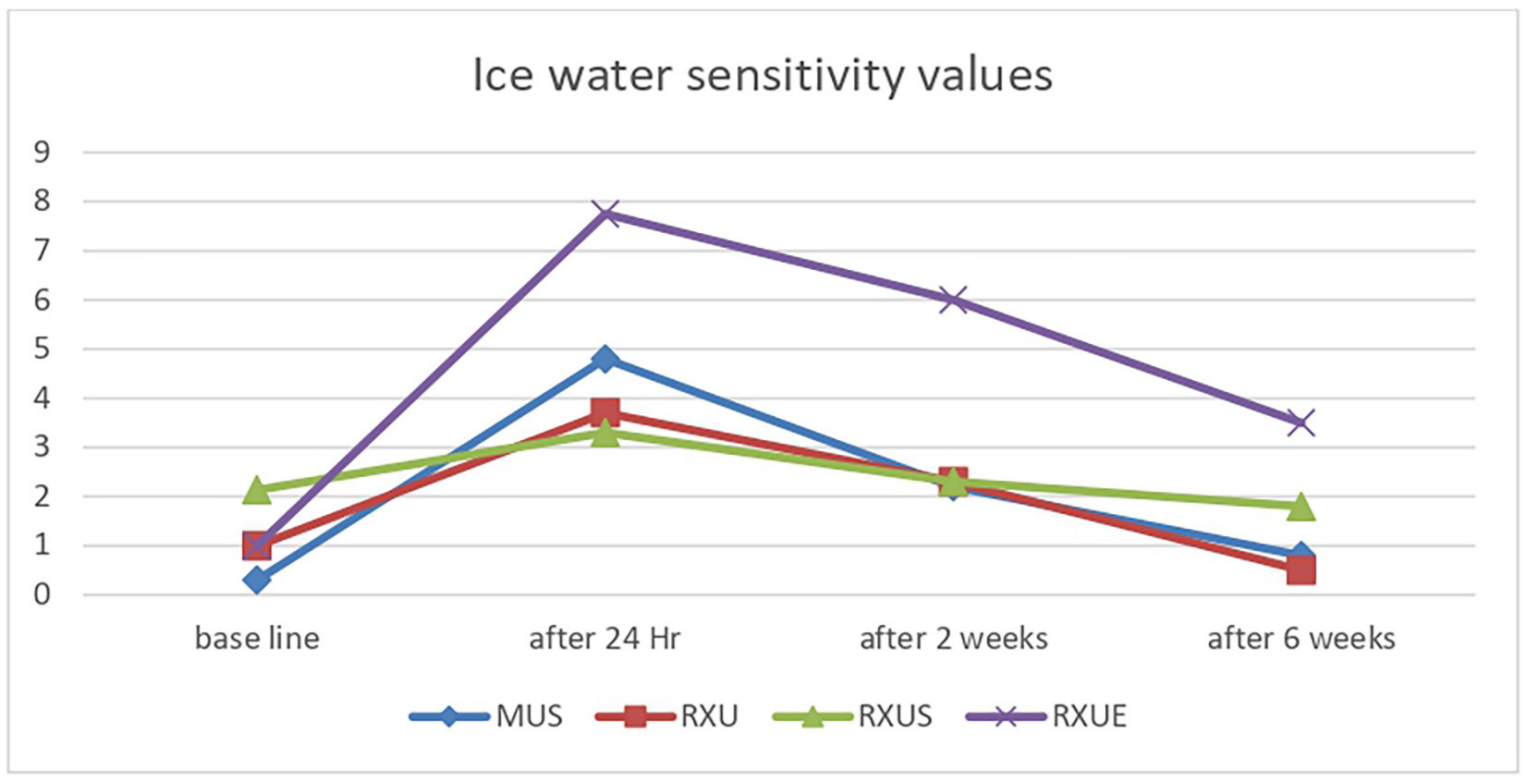
Ice water sensitivity values for used resin cements throughout period of the study using VAS.

**Figure 2:**
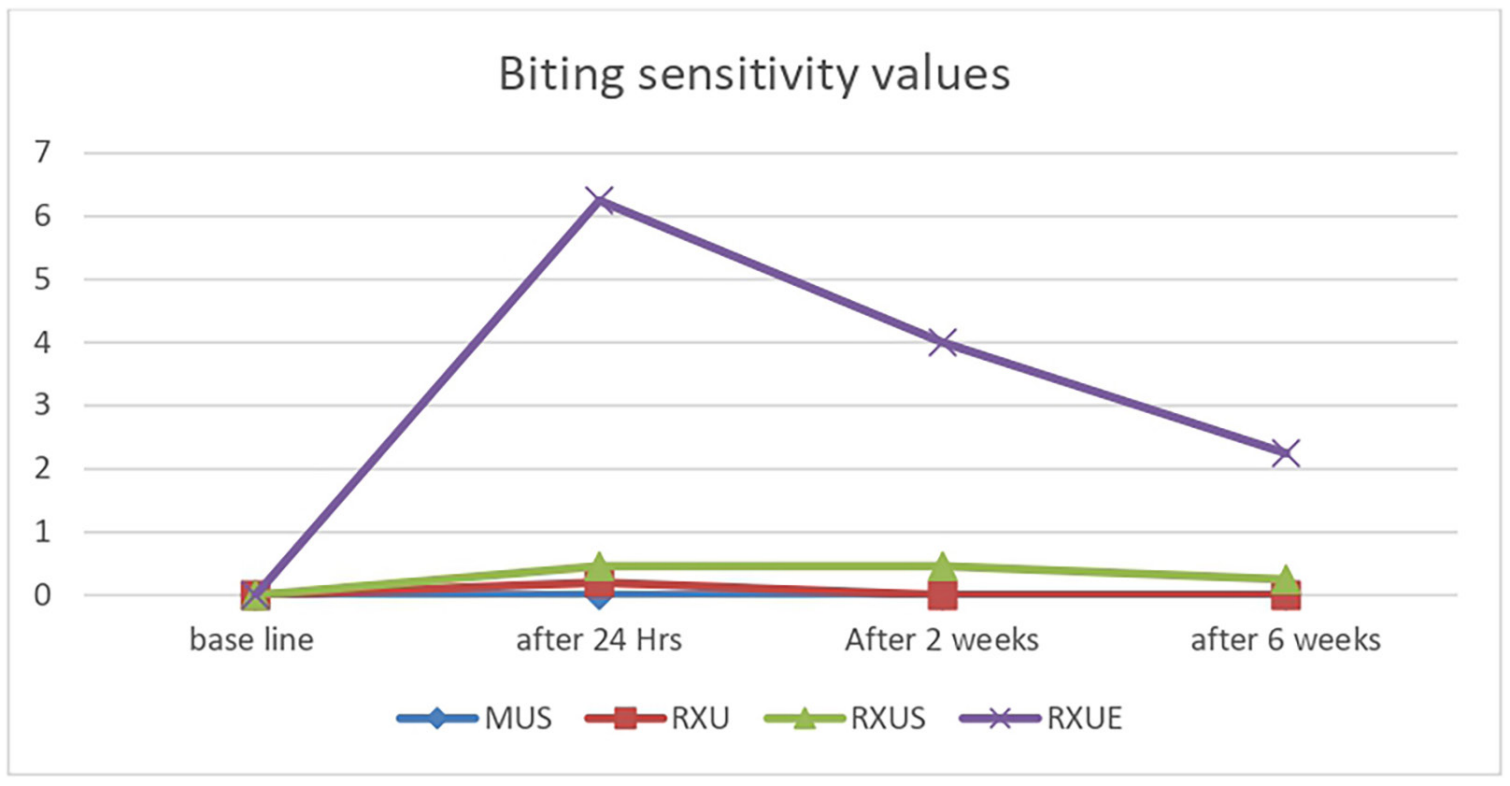
Biting sensitivity values for used resin cements throughout period of the study using VAS.

**Figure 3:**
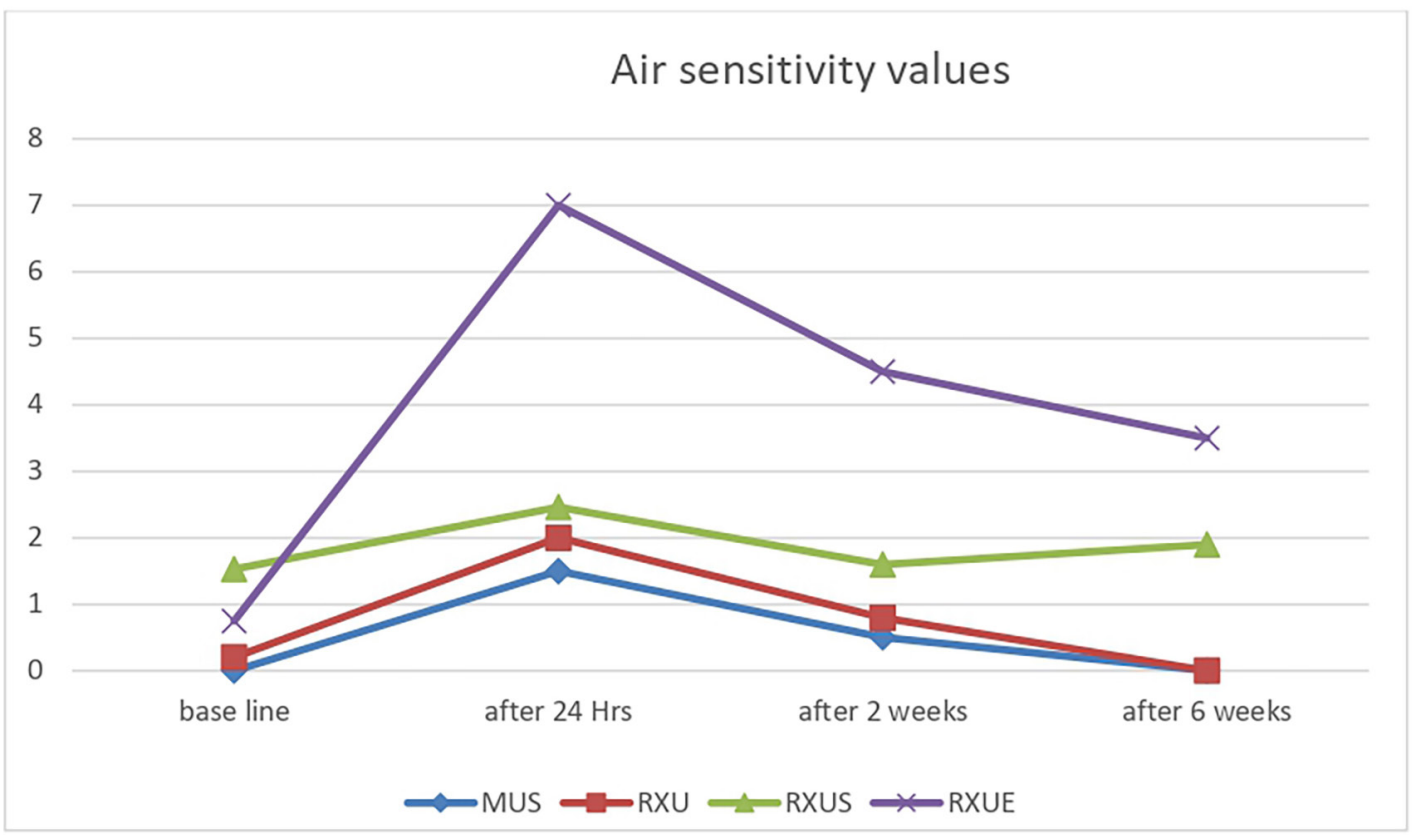
Air sensitivity values for used resin cements throughout period of the study using VAS.

In the air sensitivity test, the mean VAS score for RXU and RXUS after 24 hours showed no significant difference from the pretreatment value (*p* > 0.05). In contrast, MUS had a significantly different score at 24 hours compared to pretreatment (*p* < 0.001), though this difference was no longer significant after two weeks (*p* > 0.05). Both self-adhesive cements, RXU and MUS and resin cement with self-etch adhesive RXUS , exhibited no sensitivity to biting across all testing intervals. Sensitivity to ice water, biting, and air for the various resin cements throughout the study is illustrated in Figs. 1,2,3.

## Discussion

The null hypothesis, which proposed no difference in post-cementation sensitivity among the FPDs cemented with the four resin cements, was rejected. Self-adhesive resin cements, MUS and RXU, along with the self-etch resin cement RXUS, demonstrated significantly lower levels of post-cementation sensitivity compared to resin cement used in etch & rinse mode RXUE.

One of the factors contributing to post-cementation sensitivity is believed to be the acid-etching of dentin, which removes the smear layer and opens pathways for bacterial penetration into the pulp. Additionally, factors such as insufficient water-cooling during tooth preparation, and excessive air-drying during cementation have been identified as potential causes of pulp irritations, leading to post-operative sensitivity. In this study, experienced clinicians performed the tooth preparations, ensuring that adequate water-cooling was maintained throughout the process. Furthermore, diamond burs were replaced after every two abutment preparations to avoid using worn burs. The study’s participants, aged between 30 and 40 years, fell into a relatively younger age group, meaning the dentin maturity of the abutments was comparable across participants.

FPD abutments were chosen rather than single crowns as the study aimed to use caries-free, and restoration-free teeth, which are difficult to crown unless used as abutments for FPD. Tilted or over-erupted abutments were excluded to avoid excessive tooth reduction, which could potentially impact sensitivity results. A clinical evaluation by Johnson *et al*. (12) revealed no significant difference in the response to cold stimuli between molars and premolars, which aligns with this study’s findings.

Conventional resin cement bonding to dentin requires the removal of the smear layer, a multi-step process that can be sensitive technique (13,14).

In contrast, self-adhesive resin cements streamline the process by eliminating the requirement for additional substances. These cements contain acidic monomer methacrylate, which aid in dentin adhesion.

The variations in post-cementation sensitivity among the self-adhesive resin cements, self-etch resin cement, and the resin cement with etch-and-rinse adhesive can be explained by the distinct procedures involved in each method. In the etch-and-rinse method, the smear layer is removed, followed by conditioning and priming of the dentin in separate steps. Common errors during this process, such as over-etching, inadequate rinsing, over-drying, or incomplete solvent evaporation, can lead to a mismatch between the extent of dentin demineralization and resin penetration, potentially causing sensitivity. These issues are less likely to occur with cements that use self-etch adhesives and self-adhesive cements, as both smear layer removal and resin infiltration happen in fewer steps without rinsing or drying.

Some *in vivo* studies suggested that resin components from the adhesive may be pushed outward after acid etching, with dentin fluid movement during etching creating a moist environment which may disrupt the bonding procedure (17,18). Additionally, the increase in temperature during photo-activation of the adhesive could cause internal fluid movement, potentially carrying uncured resin via the dentinal tubules towards the pulp (9). Several researches has demonstrated that residual monomers from resin materials can exert toxic effects on cell (20,21) and *in vivo* research has revealed that resin components which come in direct contact with pulpal tissue can induce pulp inflammation (15,16). However, one histological study examining the pulp’s response to adhesive-resin cements observed that Rely-X Unicem did not form resin tags or displace cement components through the dentin (22). The cement’s specific properties, including minimal solubility and auto-neutralizing mechanism during the the setting process, were believed to inhibit further hydrolysis and the diffusion of components into the dentinal tubules. A crucial characteristic of self-adhesive resin cement is the interaction with the smear layer, this involves altering rather than completely removing it, reducing the risk of pulp irritation and post-cementation hypersensitivity.

The findings of this study align with previous research showing that self-adhesive resin cements reduce post-operative sensitivity in crowned vital teeth when compared to crowns cemented with either glass ionomer or resin-modified glass ionomer cements (23,24). Additionally, the study supports other research that found reduced post-cementation sensitivity in FPDs cemented with self-adhesive resin cements (25).

In contrast, some studies (26,27) that compared post-operative sensitivity in composite restorations using self-etch or etch-and-rinse bonding systems found no significant difference in sensitivity between the two. However, these studies often involved teeth with caries or old amalgam or composite restorations, where secondary dentin formation may have reduced dentinal tubule diameter and lessened the impact of etching and adhesives on tooth sensitivity. In the current study, sound teeth were used as abutments for FPDs, and freshly cut dentin, along with a larger surface area, may have allowed for greater penetration of etching and adhesive agents, potentially triggering an inflammatory response in the pulp and increasing post-operative sensitivity.

Study Limitations: While this clinical trial attempted to control most variables affecting sensitivity, it is inherently difficult to manage all factors in a clinical setting. Additionally, the study only included four resin cements, limiting the generalizability of the results. Future studies should include a broader range of cements.

## Conclusions

The use of self-adhesive resin cements RXU and MUS, as well as the resin cement RXUS with a self-etch adhesive, resulted in significantly lower post-cementation sensitivity in FPDs compared to the resin cement RXUE, which utilized the etch-and-rinse technique.

## Figures and Tables

**Table 1:** Means and SDs of VAS scores for cold, biting and air sensations at different time intervals for the four cements. *: denotes a statistically significant difference Vs RXUE mean within same sensation test (*p*< 0.05). #: denotes a statistically significant difference Vs pre-treatment mean (*p*< 0.05).

Test type	Cement	Pre-treatment	24 hours	2 weeks	6 weeks
Cold water	Multilink Speed	0.3 (0.52)	4.8 (2.1) *#	2.2 (1.6) *#	0.8 (1.2) *
	Rely-X Unicem	1 (0.8)	3.7 (1.8) *#	2.3 (1.4) *	0.5 (0.54) *
	RelyX-Ultimate self-etch	2.13 (0.2)	3.3* (0.3)	2.3* (0.1)	1.8 * (0.2)
	Rely-X Ultimate etch& rinse	1 (0.8)	7.75 (1.5) #	6 (2.2) #	3.5 (2.4) #
Biting	Multilink Speed	0 (0)	0 (0) *	0 (0) *	0 (0) *
	Rely-X Unicem	0 (0)	0.2 (0.4) *	0 (0) *	0 (0) *
	RelyX-Ultimate self-etch	0(0)	0.46 (0.03) *	0.46 (0.05) *	0.25 (0.05) *
	Rely-X Ultimate etch& rinse	0 (0)	6.25 (1.7) #	4 (3.4) #	2.25 (2.2) #
Air	Multilink speed	0 (0)	1.5 (1.04) *#	0.5 (0.8) *	0 (0) *
	Rely-X Unicem	0.2 (0.4)	2 (2.4) *	0.8 (0.7) *	0 (0) *
	RelyX-Ultimate self-etch	1.53 (0.2)	2.46 (0.3) *	1.6 (0.2) *	1.9 (0.2) *
	Rely-X Ultimate etch & rinse	0.75 (0.5)	7 (1.8) #	4.5 (2.9) #	3.5(2.4) #

## Data Availability

The datasets used and/or analyzed during the current study are available from the corresponding author.
